# Development and Characterization of a Semi-Solid Dosage Form of Meglumine Antimoniate for Topical Treatment of Cutaneous Leishmaniasis

**DOI:** 10.3390/pharmaceutics11110613

**Published:** 2019-11-15

**Authors:** Diana Berenguer, Lilian Sosa, Magdalena Alcover, Marcella Sessa, Lyda Halbaut, Carme Guillén, Roser Fisa, Ana Cristina Calpena-Campmany, Cristina Riera

**Affiliations:** 1 Department of Biology, Health and Environment, Laboratory of Parasitology, Faculty of Pharmacy and Food Sciences, University of Barcelona, 08028 Barcelona, Spain; 2 Department of Pharmaceutical Technology and Physicochemistry, Faculty of Pharmacy and Food Sciences, University of Barcelona, 08028 Barcelona, Spain

**Keywords:** *Leishmania infantum*, cutaneous leishmaniasis, meglumine antimoniate, Sepigel 305^®^, topical treatment

## Abstract

Cutaneous leishmaniasis (CL) is treated with painful intralesional injections of meglumine antimoniate (MA). With the aim of developing an alternative topical treatment for CL, a gel-based formulation with 30% MA was prepared and its physicochemical properties, stability and rheological behavior were studied. The following were assessed: drug release on propylene hydrophilic membranes ex vivo human skin permeation, tolerance in healthy volunteers, cytotoxicity in three cell lines and anti-leishmanial activity against *Leishmania infantum* promastigotes and amastigotes. The MA gel formulation was found to have suitable pH, and good spreadability and stability. Low quantities of pentavalent antimony (Sb^V^) were observed in release and permeation tests, whereas retention was high in both non-damaged and damaged skin (71,043.69 ± 10,641.57 and 10,728 ± 2254.61 µg/g/cm^2^ of Sb^V^, respectively). The formulation did not have a toxic effect on the cell lines, and presented lower Sb^V^ IC_50_ values against amastigotes (15.76 ± 4.81 µg/mL) in comparison with the MA solution. The high amount of drug retained in the skin and the Sb^V^ IC_50_ values obtained suggest that this semi-solid dosage form has potential as an alternative treatment of CL.

## 1. Introduction

Leishmaniasis is caused by protozoan parasites, including more than 20 *Leishmania* species, and is transmitted through the bites of infected female phlebotomine sandflies. It is a neglected tropical disease that is prevalent in low-income countries [1] and is endemic in 98 countries with tropical, temperate and mild temperate climates, with an estimated 12 million people infected by the disease. There are three types of leishmaniasis: cutaneous (CL), mucocutaneous (MCL) and visceral (VL). The most common form of the disease worldwide is CL, with 0.6–1 million new cases estimated to occur annually [2]. In the Mediterranean area, *Leishmania infantum* is the main species responsible for CL and VL [3]. Depending on the *Leishmania* species involved, manifestations of CL range from self-resolving ulcerative lesions, which may take up to two years to heal, to painful open wounds that leave permanent scars or even disseminate and affect other areas of the body [4].

Treatments proposed for CL depend on the infecting species, patient immunity, as well as the number, sizes and location of lesions, but none of them are universally effective [5]. CL cases presenting small and localized lesions are usually treated with pentavalent antimony salts, sodium stibogluconate or meglumine antimoniate (MA). These first-line drugs are administered by intralesional infiltrations requiring multiple injections that cause acute pain to the extent that patients are often unable to complete the treatment [6]. As it is a common mistake when talking about MA treatments for leishmaniasis to confuse the therapeutic dose that refers to MA with the amount of pentavalent antimony (Sb^V^), henceforth we will refer all the concentrations to the amount of Sb^V^ since it is the main active ingredient.

Consequently, efforts have been made to develop alternative drugs and forms of administration.

According to the World Health Organization (WHO), topical and local therapies with less systemic toxicity (thermotherapy, cryotherapy, paromomycin ointment, local infiltration with antimonials) are good treatment options for patients with a low number of small lesions [7].

Topical treatments have many advantages: they are easy to administer, do not require hospitalization or other infrastructure, favor patient commitment, are low cost and have fewer toxic side effects [8,9,10].

A widely used treatment is the application of 15% paromomycin ointments in association with 12% methylbenzethonium chloride twice a day for 20 days, although results are variable, and patients experience significant irritation, inflammation, pain and pigmentation [11,12]. Cure rates of around 80% have been obtained in some clinical trials [13,14], whereas others report lower efficiency [15]. Liposomes and a nanogel formulation containing paromomycin have also been developed [6,16,17].

Alternatively, topical conjugates with amphotericin B were reported to penetrate the skin in a murine model [18]. However, the cure rates in a phase II study with topical 3% amphotericin B cream for uncomplicated CL were low and its clinical development as a therapeutic option was discontinued [19].

Clinical trials have also been performed with a topical formulation of miltefosine, but no efficacy against CL has been proven [10] despite experimental trials in mice reporting a reduction in lesion size and full recovery, albeit with relapse [20].

Regarding antimonials, there are few data available on semi-solid dosage forms for topical administration. The development and characterization of liposomes loading sodium stibogluconate and MA has been described [21,22,23].

Sepigel 305^®^, a product used in gels and gel-creams, consists of a gelling agent (polyacrylamide), a non-ionic emulsifier (polyoxyethylene 7 lauryl ether) and a fatty oil (isoparaffin). Simple gel formulations containing Sepigel 305^®^ have medium to high viscosity [24], a degree of cooling power, evanescent properties and optimal dermocosmetic qualities. They also allow the incorporation of both hydrophilic and lipophilic substances, and previous studies on different drugs have reported that formulations containing this polymer have good stability [24,25,26].

In this context, the use of hydrogels could be a promising strategy to deliver MA for the management and healing of CL. The aim of the present study was therefore to design and develop a gel-based formulation containing MA for CL treatment. Physico-chemical parameters were monitored to determine stability over time. Human skin permeation and retention ex vivo and tolerance in vivo were tested to optimize a new dosage system of the drug with fewer side effects. Cytotoxic effects and leishmanicidal activity in vitro were assayed in promastigotes and intracellular amastigotes of *L. infantum*.

## 2. Materials and Methods

### 2.1. Materials

Meglumine antimoniate was obtained from Acros Organics (Thermo Fisher Scientific, Waltham, MA, USA). Gentamicin was acquired from Sigma-Aldrich (Darmstadt, Germany), and Sepigel 305^®^ from Acofarma (Barcelona, Spain). Distilled water utilized in the experiments was obtained from a Mili-Q^®^ Plus System (Millipore Co., Burlington, MA, USA).

### 2.2. Preparation of the Gel

Briefly, a gel-based formulation containing 30% of MA (with 31.6% Sb^V^ *w*/*w*) was prepared. MA was dissolved in water with 0.5% of gentamicin. Sepigel 305^®^ was added under continuous stirring to obtain the gel formulation at a concentration of 4.5%.

### 2.3. Physicochemical Characterization of the MA Gel

#### 2.3.1. Morphological Analysis

To analyze its morphology, the MA gel was dried over a period of 7 days using a vacuum desiccator. Once totally dried, 0.1 g was coated with carbon as a conductive agent. The internal structure of the gel was examined by scanning electron microscopy (SEM) using a JEOL J-7100FE (Peabody, MA, USA).

#### 2.3.2. Swelling and Degradation Tests

A gravimetric method was used to obtain the swelling ratio (SR) and to test degradation, which was represented as the percentage of weight loss (WL). Dried and fresh MA gel were used to carry out the swelling and degradation tests, respectively. In both experiments the MA gel was incubated in PBS (pH = 5.5) at 32 °C for 20 min. Samples (*n* = 6) were removed and weighed after blotting the surface water at predetermined time intervals of 3 min. The SR was calculated using the following equation and expressed by kinetic modeling:

(1) $$\mathrm{SR}=\frac{Ws-Wd}{Wd}$$

where *Ws* is the weight of the swollen MA gel at 3 min intervals for 20 min and *Wd* is the weight of dried gel.

WL was calculated following the equation and expressed by kinetic modeling:

(2) $$\mathrm{WL}\;\left(\%\right)=\frac{Wi-Wd}{Wi}\;\times \;100$$

where *Wi* is the initial weight of the MA gel and *Wd* is the gel weight each 3 min.

#### 2.3.3. Water Loss Due to Drying

Water loss was evaluated by weighing 1 g of fresh MA gel, which was placed in a vacuum desiccator for 7 days until the weight was constant.

#### 2.3.4. Porosity Study

The porosity percentage (P) was calculated by a solvent displacement method, which consisted of immersing the previously dried MA gel in absolute ethanol for 2 min and then weighing it after the excess ethanol on the surface was blotted. The porosity percentage was calculated using the following equation:

(3) $$P\;\left(\%\right)=\;\frac{W2\;-\;W1}{\rho \;\times \;V}\;\times \;100$$

where *W*1 represents the weight of the dried MA gel, *W*2 stands for the weight of the MA gel after being immersed in ethanol, ρ is the density of absolute ethanol and *V* is the volume of the gel.

### 2.4. Stability Studies

The MA gel was stored at room temperature (RT), 4 °C and 37 °C. The pH values of the formulation were measured with universal test paper (Filter-Lab^®^) by direct spread of the samples (*n* = 3). Appearance was visually inspected, and pH was measured after 30 days and after 6 months.

Sb^V^ was quantified by ICP-OES (Inductively coupled plasma-optical emission spectrometry, Perkin Elmer Elan 6000, Waltham, MA, USA) after previous treatment of the samples with HNO_3_ and H_2_O_2_ in a microwave digester at 220 °C for 72 h. The drug content was evaluated at the beginning and end of the 6-month experiment.

### 2.5. Rheological Studies

The rheological measurements were performed in duplicate 24 h after gel preparation using a rotational rheometer (Thermo Scientific HaakeRheostress 1, Thermo Fischer Scientific, Karlsruhe, Germany) equipped with a cone plate set-up (0.105 mm gap between cone and plate) with a fixed lower plate and a mobile upper cone Haake C60/2° Ti (60 mm diameter, 2° angle). The rheometer was connected to a temperature control ThermoHaake Phoenix II + Haake C25P (Thermo Fischer Scientific, Waltham, MA, USA) and a computer provided with the HaakeRheowin^®^ Job Manager v. 4.0 (Thermo Fischer Scientific, Waltham, MA, USA) to carry out the tests and HaakeRheowin^®^ Data Manager v.4.0 (Thermo Fischer Scientific) to carry out the analyses of the obtained data. Viscosity curves (η = f($\dot{\gamma }$)) and flow curves (τ = f($\dot{\gamma }$)) were recorded at 25 °C. The shear rate ramp program included: a ramp-up period from 0 to 50 s^−1^ for 3 min; constant shear rate period of 50 s^−1^ for 1 min; and a ramp-down period from 50 to 0 s^−1^ for 3 min. The data from the flow curves (τ = f($\dot{\gamma }$)) were fitted to different mathematical models equations: Newton, Bingham, Ostwald-de-Waele, Herschel-Bulkley, Casson and Cross [27]. Best fit of mathematical models was based on the correlation coefficient value (r). The viscosity mean value (Pa·s) was determined from the constant share section at 50 s^−1^.

### 2.6. Spreadability Test

The spreadability of MA gel and MA solution was determined in triplicate as follows: 0.5 g of formulation and 150 µL of MA solution were placed within 1 cm diameter circles previously marked on a glass plate, and also on a plastic plate. A series of weights (5, 10, 20, 50 and 100 g) were successively added and allowed to rest for 2 min each at RT. The diameters (cm^2^) of the circle spreads were measured and recorded as comparative values.

### 2.7. In Vitro Release Studies

Vertical Franz diffusion cells (FDC 400; Crown Glass, Somerville, NJ, USA) were used to assay the MA release with hydrophilic polypropylene membranes (GH Polypro, Life Sciences). The receptor medium was water at 32 ± 0.5 °C under stirring at 600 rpm in sink conditions. The experiment was performed with a diffusion area of 0.64 cm^2^. Small quantities of MA gels (0.3 g) or MA solution (300 µL) were added to the donor compartment. At the end of the study at 55 h, the amount of Sb^V^ in the receptor compartment was analyzed by ICP-OES (Perkin Elmer Elan 6000). Results were reported as the mean ± SD of five replicates.

### 2.8. Ex Vivo Permeation Studies

Ex vivo permeation tests were performed as described in Section 2.7 using damaged and non-damaged human skin samples with a thickness of 400 µm from a single donor. The skin was damaged using the tape stripping technique, which involves repeated application of adhesive tapes to the skin surface to remove stratum corneum layers. For the assay, adhesive tapes were applied to the skin 7 times to damage the skin but not remove it completely. Then 0.3 g of MA gel was placed in the donor compartment. After 27 h the quantity of Sb^V^ was determined by ICP-OES in five replicates.

To determine the amount of drug retained in the epidermal tegument, the skin membranes were detached from the Franz cells, cleaned with gauze soaked in 0.05% sodium dodecylsulphate solution and washed in distilled water. The area of permeation was then cut and weighed. Finally, the Sb^V^ retained was extracted in an ultrasound bath with water for 30 min. The resulting solutions were measured by ICP-OES for quantification.

The Plastic Surgery Department of Barcelona-SCIAS Hospital (Barcelona, Spain) provided the human skin from the abdominal region of a healthy woman, and the Bioethics Committee of the same hospital approved the experimental protocol (reference number: BEC/001/16; date: 15 January 2016). Written informed consent forms were provided. Human skin was dermatomed (GA630 dermatome, Aesculap, Tuttlingen, Germany) into pieces with a thickness of 400 μm [28]. The integrity of skin samples was assessed in triplicate by measuring the trans-epidermal water loss (TEWL) values using a DermaLab^®^ module (Cortex Technology, Hadsund, Denmark).

### 2.9. In Vivo Tolerance Study

Ten female volunteers with healthy skin between 25 and 35 years old participated in the study. The study was approved by the Ethics Committee of the University of Barcelona (reference number: IRB00003099; date: 20 March 2018) following the guidelines of the Declaration of Helsinki [29] and all volunteers signed written informed consent forms. Skin-care cosmetics were not permitted on the test areas for two days prior to the study. Volunteers stayed in the test room for at least 30 min prior to the measurements. Measurements were performed before applying the gel (to establish the baseline readings) and 15 min, 1 h and 2 h after the application of 0.5 g on the flexor side of the left forearm.

Trans epidermal water loss (TEWL), referring to the total amount of water vapor lost through the skin was measured by a Tewameter^®^ TM 300 (Courage-Khazaka electronic GmbH, Cologne, Germany). The stratum corneum hydration (SCH) was determined using a Corneometer^®^ CM 825 (Courage-Khazaka electronic GmbH). Measurements were performed according to international guidelines [30].

### 2.10. Parasite Strains and Cultures

The *Leishmania infantum* strain MHOM/ES/2016/CATB101 isolated from an individual with CL from Mallorca (Spain) was used. Promastigotes were cultured at 26 °C in Schneider insect medium, pH 7.0, with 20% heat-inactivated fetal calf serum, 25 µg/mL gentamicin solution (Sigma G-1397, St. Louis, MO, USA), and 1% penicillin (100 U/mL)-streptomycin (100 mg/mL) solution (Sigma P4333).

### 2.11. In Vitro Cytotoxicity Assay

In order to study the cytotoxicity of the MA gel and the gel excipients, 5 × 10^4^ cells/mL of the cell lines RAW 264.7, J774A.1 and HaCaT were cultured in 96-well plates (Costar 3595). Serial dilutions of the gel in RPMI-1640 medium with 10% heat-inactivated fetal calf serum and 1% penicillin (100 U/mL)-streptomycin (100 mg/mL) were added. After 24 h of incubation at 37 °C in a 5% CO_2_ atmosphere, WST-1 reagent (Roche Diagnostics GmbH, Mannheim, Germany) was added to each well and the plate was incubated in the same conditions for 4 h. The absorbance of the samples was measured at 450 nm using a spectrophotometer (Multiskan EX, Thermo Electron Corporation, Shanghai, China). The concentration inhibiting 50% of cell viability (CC_50_) was determined by linear regression analysis and experiments were performed in triplicate [31].

### 2.12. In Vitro Anti-Leishmanial Activity against Promastigotes

The activity of MA gel, gel alone and MA solution was studied on promastigotes in microtiter plates (Costar 3595). Serial dilutions of the gel in Schneider medium were seeded, and then 10^6^ promastigotes/mL in their logarithmic growth phase were added to each well and incubated at 26 °C for 48 h. Growth was measured through acid phosphatase and the optical density was determined at 405 nm with a spectrophotometer (Multiskan EX, Thermo Electron Corporation). The concentration inhibiting 50% of parasite growth (IC_50_) was determined by linear regression analysis of the minimum squares of parasitic growth versus the logarithm of the drug concentration with 95% confidence interval. Experiments were performed in triplicate [32].

### 2.13. In Vitro Anti-Leishmanial Activity against Intracellular Amastigotes

J774A.1 cells at a concentration of 5 × 10^4^ cells/mL were cultured in an eight LabTek chamber slide system (Nunc^®^). After 24 h, late stationary phase promastigotes from a 5-day culture in RPMI-1640 complete medium with 10% heat-inactivated fetal calf serum and 1% penicillin (100 U/mL)-streptomycin (100 mg/mL) were added at a concentration of 5 × 10^5^ cells/mL and incubated for 24 h at 37 °C in 5% CO_2_ atmosphere. Free promastigotes were removed by washing. RPMI-1640 complete medium with serial dilutions of the gel was added to each well and incubated at 37 °C in 5% CO_2_ atmosphere for 48 h. Infected cells were washed and slides were stained with Giemsa. Drug activity was evaluated by counting the number of infected cells by examining 300 macrophages in triplicate [33].

### 2.14. Statistical Analysis

Experimental data obtained were analyzed by one-way parametric analysis of variance (ANOVA), followed by a multiple comparison Tukey test. A *p* < 0.05 indicated the differences were statistically significant. Prism^®^ V. 5 (GraphPad Software Inc., San Diego, CA, USA) was used for calculations.

## 3. Results

### 3.1. Physicochemical Characterization of the MA Gel

**Figure 1:**
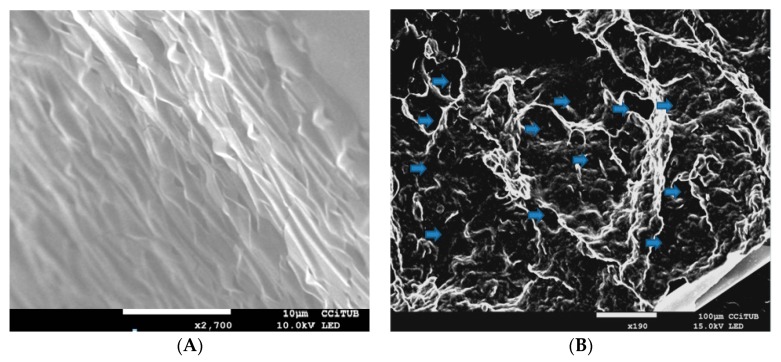
SEM images of the meglumine antimoniate (MA) gel, (**A**) laminar disposition (2700×) and (**B**) porous structure (190×).

As shown in Figure 1A,B, SEM revealed a laminar disposition in the gel formulation. The layers were regularly organized and a porous structure was evident.

**Figure 2:**
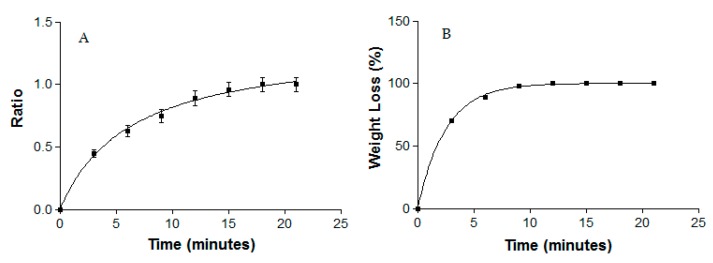
(**A**) Swelling ratio and (**B**) percentage of weight loss in the degradation of MA gel.

The swelling process of MA gel followed a hyperbolic model, which was represented by the kinetic constants k = 0.64 min^−1^ (r^2^ = 0.9986) (Figure 2A). The degradation of MA gel was completed in 15 min and followed a one phase exponential model with a kinetic constant k = 0.01 min^−1^ (r^2^ = 0.9995) (Figure 2B), and the P percentage of MA gel was ~89.37 ± 0.15%.

After 7 days of drying the gel, the sample showed 22 ± 2.13% loss of water content.

### 3.2. Drug Quantification and Stability Studies

**Figure 3:**
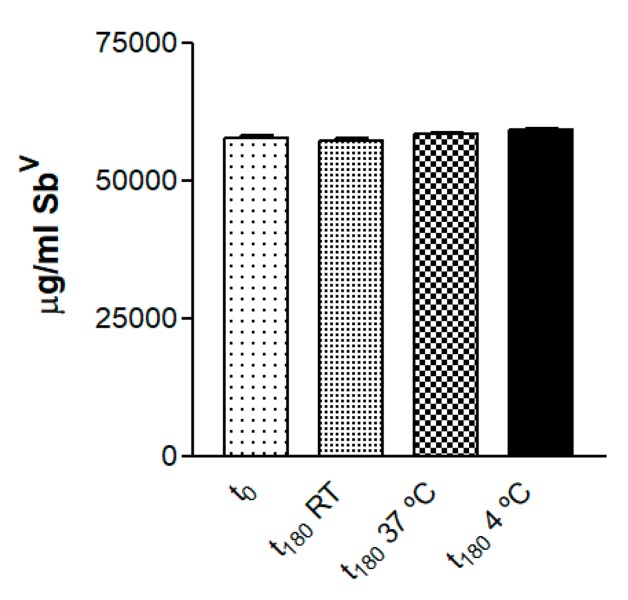
Concentration of Sb^V^ in the gel at different storage temperatures at the beginning of the experiment and 6 months later.

Determination of the drug content revealed 72.7% entrapment efficiency of Sb^V^ in the gel formulation. The concentration of Sb^V^ in the reference solution and in the gel over time is summarized in Table 1. The Sb^V^ content of the gel samples at the tested temperatures did not present statistical differences (*p* > 0.05) after 6 months (Figure 3).

The MA gel had a pH value of 5–6 at the beginning and end of the experiment.

When freshly prepared, the gel formulation was a semi-transparent white with homogeneous appearance without precipitates. After 30 days at RT and 4 °C, the formulation had the same appearance as at the beginning of the experiment, whereas at 37 °C it turned yellow. After 6 months at 4 °C it looked the same as at the beginning of the experiment, while at RT it was a semi-transparent light yellow and at 37 °C a semi-transparent dark yellow.

### 3.3. Rheological Studies

**Figure 4:**
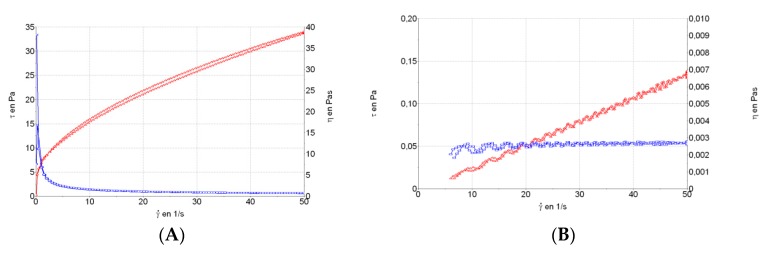
Rheological behavior of (**A**) MA gel and (**B**) MA solution.

The MA gel formulation exhibited pseudoplastic behavior (Cross model; r^2^ = 0.9997) (Figure 4A). Viscosity at 50 s^−1^ was 677.8 ± 1.76 mPa·s, and it was non-thixotropic. The MA solution showed Newtonian behavior (Newton model; r^2^ = 0.9967) (Figure 4B) and viscosity at 50 s^−1^ was 2.67 ± 0.06 mPa·s.

### 3.4. Spreadability Test

**Figure 5:**
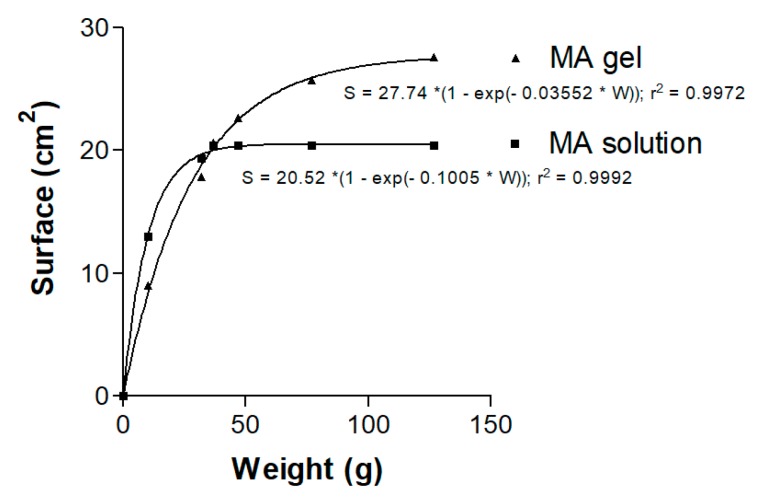
Spreadability graphic and first-order kinetic model equations for the MA gel and MA solution.

The equation that best fitted the spreadability behavior of the MA gel and solution followed a first-order kinetic model [S = S_max_ × (1 − exp(−k × W))], where S is the extension surface (cm^2^), S_max_ is the maximum extension surface (cm^2^), k is the kinetic constant (g^−1^) and W is the weight added (g). The resulting graphics and equations are shown in Figure 5. Spreadability of the MA gel increased as more weight was applied, until a maximum value was reached.

### 3.5. In Vitro Release Studies

**Figure 6:**
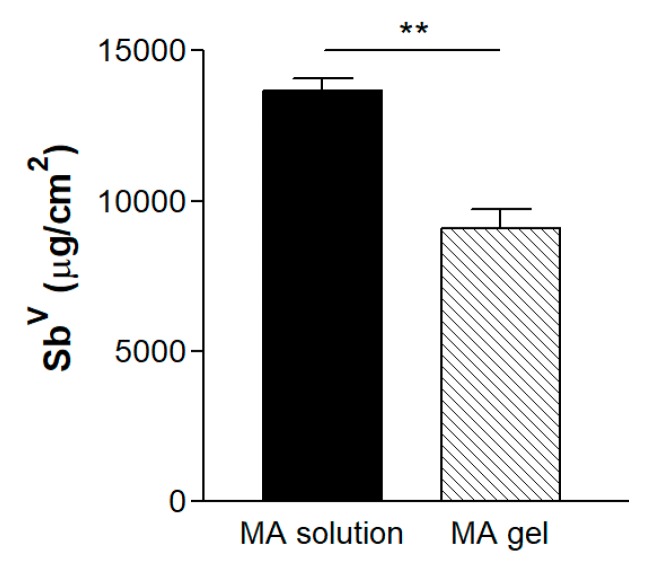
Flux of Sb^V^ released after 55 h through hydrophilic polypropylene membranes (** *p* < 0.01).

After 55 h the amount of Sb^V^ released in the receptor compartment through the hydrophilic polypropylene membranes was 13,646.53 ± 960.63 µg/cm^2^ of Sb^V^ for MA solution and 9081.13 ± 1446.20 µg/cm^2^ of Sb^V^ for the gel, corresponding to a release of 32.3 ± 3.56% and 21.5 ± 3.43%, respectively, from the total amount seeded. The differences (*p* < 0.01) between the Sb^V^ released from the solution and the gel are statistically significant (Figure 6).

### 3.6. Ex Vivo Permeation Studies

Table 2 summarizes the amount of Sb^V^ permeated (µg/cm^2^) through the skin and the amount retained (µg/g/cm^2^). In both permeation and retention assays, higher Sb^V^ values were obtained for non-damaged than damaged skin when testing the reference solution and the MA gel. The percentage of Sb^V^ in the reference solution and MA gel that permeated through the healthy skin was 7.03 ± 1.23% and 2.89 ± 0.83%, and through the damaged skin 0.29 ± 0.51% and 1.84 ± 0.65%, respectively.

### 3.7. In Vivo Tolerance Study

**Figure 7:**
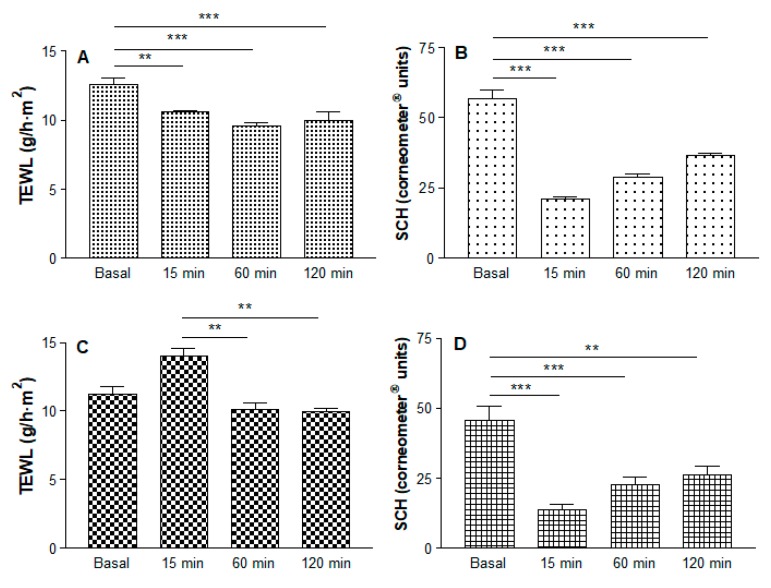
Measurements of biomechanical parameters of skin for 2 h after application of the gel and solution. (**A**) Change in trans-epidermal water loss (TEWL) and (**B**) Stratum corneum hydration (SCH) values after application of MA solution. (**C**) Change in TEWL and (**D**) SCH values after application of MA gel (** *p* < 0.01; *** *p* < 0.001).

The results for the TEWL and SCH are shown in Figure 7. The MA solution decreased the TEWL with statistically significant differences for all of the experiment times (Figure 7A). The MA gel increased the TEWL after 15 min, and basal values were almost recovered after 1 h, with statistically significant differences between 15 min and 60 min, and between 15 min and 120 min (Figure 7C). As the graph shows, significant differences in SCH values were found when comparing baseline values with all the experiment times for both formulations, revealing the dehydration of the stratum corneum and its slow gradual recovery (Figure 7B,D).

### 3.8. In Vitro Cytotoxicity Assay

**Figure 8:**
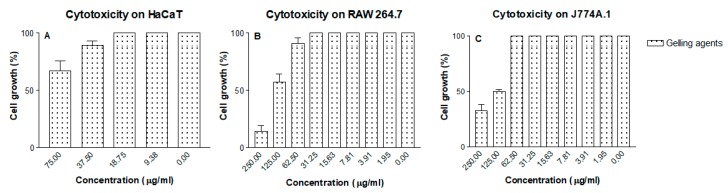
Cytotoxicity of the gelling excipients in cell lines (**A**) HaCaT, (**B**) RAW 264.7 and (**C**) J774A.1.

**Figure 9:**
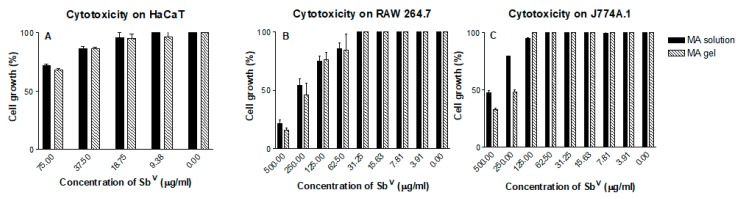
Cytotoxicity of MA solution and MA gel in cell lines (**A**) HaCaT, (**B**) RAW 264.7 and (**C**) J774A.1.

Figure 8 and Figure 9 show the cytotoxic effects on the cell lines assayed. No cytotoxicity was observed in the keratinocyte cell line HaCaT at the ranges of dilution tested. The gel formulation with and without MA showed cytotoxicity in the RAW264.7 and J774A.1 cell lines only at the highest concentrations of the gelling excipients and Sb^V^ assayed. The CC_50_ of the MA gel and the gel alone are summarized in Table 3 and Table 4.

### 3.9. Anti-Leishmanial In Vitro Activity against Promastigotes

The effects of the MA solution and MA gel are summarized in Table 3, and Table 4 shows the effect of the gel without the drug. Neither the MA gel nor the gel alone were able to inhibit the growth of *L. infantum* promastigotes, with Sb^V^ IC_50_ values of 633.15 ± 43.26 µg/mL (0.70% of gel) for the MA gel and IC_50_ values greater than 375 µg/mL (0.71% of gel) for the gel alone.

### 3.10. Anti-Leishmanial In Vitro Activity against Intracellular Amastigotes

**Figure 10:**
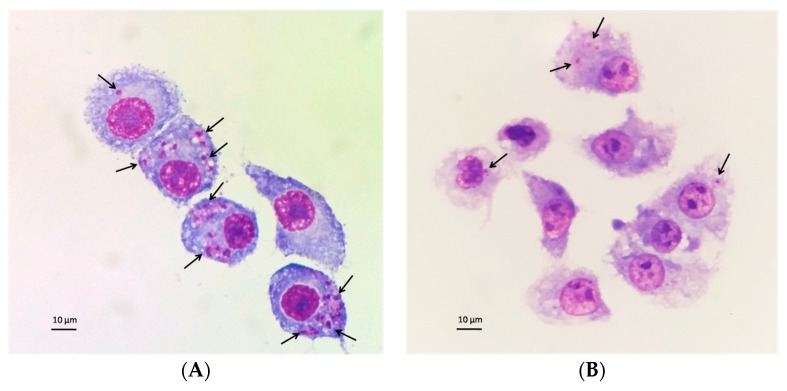
Effect of the MA gel on the infection of J774A.1 macrophages with *L. infantum*. (**A**) Infected and untreated control cultures. (**B**) Cultures treated with 15.63 µg/mL of Sb^V^ of the MA gel. Amastigotes are indicated by arrows.

The activity of the MA solution and MA gel is summarized in Table 3. In the amastigote assay the MA gel presented lower Sb^V^ IC_50_ values (15.76 ± 4.81 µg/mL) in comparison with the MA solution (57.35 ± 2.76 µg/mL). As shown in Table 4, the gelling excipients showed some activity against the amastigotes of *L. infantum*. Figure 10 shows the images of control infected macrophages and infected macrophages treated with MA gel.

## 4. Discussion

The development of an effective topical treatment for CL is an interesting but difficult challenge. One of the main problems in topical administration is that *Leishmania* amastigotes are localized within dermal macrophages, in the papillary dermis beneath the epidermis [34]. Thus, after the drug is released from the vehicle, it must cross the stratum corneum in the skin barrier and reach the macrophages to deliver the pharmacological action.

In this study, we developed a Sepigel 305^®^-based formulation for the dermal delivery of MA with qualities that could enhance the activity of MA against CL with minimal side effects.

The water content in the MA gel formulation represented almost a quarter of its composition. Degradation was constant over time, indicating it did not depend on the remaining polymer concentration, and the process was completed in 15 min. The very high porosity percentage of the MA gel was corroborated by SEM images, which also revealed a regular laminar disposition in the superficial structure of the gel layers. The porous structure, similar to that described by Dong et al. (2015) [35], allows the drug to be released when applied on membranes or skin.

The pH plays an important role in the stability of gels as well as in skin tolerability, and should be between 5 and 6 for skin formulations [26]. The tested formulation maintained a pH of 5–6 throughout the experiment, indicating biocompatibility for skin applications, with minimal risk of irritation or bacterial or fungal infection [36]. Anchisi et al. (2001) studied the stability of a wide range of formulations containing the same gel base over 6 months, and observed minimal changes in the pH [24].

At the beginning of the experiment the quantity of Sb^V^ found in the gel was 57,936.62 µg/mL, 27.3% less than in the reference solution consisting of MA in water. This reduction may be explained by incomplete digestion during the analytical procedure, considering the high quantity of organic material, which may mask the actual amount of Sb^V^. The stability test indicated that the amount of Sb^V^ in the gels remained constant for 6 months at all the tested storage temperatures, although the organoleptic characteristics varied. Samples stored at 4 °C retained the same visual properties throughout the 6 months (a homogeneous semi-transparent white appearance), whereas samples stored at RT turned pale yellow in the third month, and at 37 °C they turned yellow after the first month. This change in color may be due to meglumine oxidation with increasing temperature. Gel homogeneity was observed in all samples, without any biphasic formation.

When comparing different topical formulations, Bilia et al. (2006) found the best long-term stability with polyacrylamide, C13-14 isoparaffin and laureth-7 [25]. Similar results were reported by Anchisi et al. (2001), who observed that samples containing Sepigel 305^®^ did not polymerize at high temperatures because of the ability of polyacrylamide to trap free water; organoleptic features of the formulations were also stable throughout the six-month study period [24].

The rheological properties of topical semi-solid dosage forms are important because they influence skin spreadability, adhesion and retention in the application site [21]. The rheological analysis indicated that the MA gel is a pseudoplastic fluid, like most gels reported in the literature, which is ideal for topical administration [37]. As such, its main characteristic is decreased viscosity when the deformation speed is increased, resulting in a good spreading capacity. The pseudoplastic behavior is determined by the cross linking of the polymer. The MA solution showed Newtonian behavior due to its predominant water composition and very low viscosity, which makes it difficult to avoid spilling or to maintain skin adhesion after application.

Spreadability, which is considered a quality parameter for skin formulations, was lost in the MA solution at 37 g, but continued to increase in the more viscous MA gel until 127 g, indicating its ease of application (see rheograms in Figure 4A,B).

The profile of drug release from the delivery vehicle can be useful for predicting in vivo behavior of the product. At 55 h, drug release from the formulation was incomplete: only 32.3 ± 3.56% of Sb^V^ from the MA solution and 21.5 ± 3.43% from the MA gel had been released. This was probably due to the large amount of sample located in the donor compartment, the slow release of the drug from the gel, and the chemical binding of the meglumine.

In addition, the permeation study performed with damaged and healthy human skin showed very low amounts of Sb^V^ in the receptor compartment at 27 h for both the MA solution and MA gel. The percentage of permeation was higher through healthy skin than damaged skin with both vehicles. In the retention study, the drug in the skin was determined in terms of µg of Sb^V^ per g and cm^2^ of skin. Higher Sb^V^ retention values were obtained for the MA gel when the skin was undamaged, probably due to better functioning and ability to absorb more drug. The same behavior was observed for the MA solution, although a lower amount of Sb^V^ was detected. Similar results were obtained by Dar et al. (2018), who tested nano-deformable liposomes with sodium stibogluconate and the same liposomes in a Carbopol gel on rat skin, and observed a low percentage of Sb^V^ permeated into the receptor compartment and a high percentage was retained in the skin [21]. Other studies with liposomes encapsulating MA, MA combined with oleic acid and MA plus stearylamine also showed low permeation of Sb^V^ across the skin barrier and high retention in mouse skin [22,23]. Bilia et al. (2006) found the highest amounts of sesquiterpenes retained in stripped skin when using a mixture of polyacrylamide, C13-14 isoparaffin and laureth-7 and other excipients, since the level of lipophilicity enhanced the permeation compared with other topical delivery systems [25].

Most substances penetrate the skin through the transepidermic route, mainly across the spaces between cells [38]. The level of penetration depends essentially on two coefficients: that of the vehicle-skin partition and the diffusion. Small molecules are able to cross the skin more easily [39], while highly lipophilic molecules can be retained in the stratum corneum [40]. The drug of our formulation may cross the skin through the intercellular route, as MA is a small molecule (MW: 365.98 g/mol), thereby allowing proper diffusion despite its hydrophilicity. In the permeation experiments, we found lower Sb^V^ values in the damaged skin than when it was not. This indicates that stratum corneum plays an important role as a drug reservoir that allows a slow release, as we could detect Sb^V^ in the receptor compartment. Regarding the new formulation, the low permeation of Sb^V^ to the receptor compartment through the skin barrier, and retention of sufficient quantities of Sb^V^ in the damaged skin after 27 h suggests the drug could remain in the skin layers where leishmania parasites are located.

In relation to biomechanical properties, the MA solution maintained TEWL values below 12 g/h·m^2^, which is considered normal, and the integrity of the stratum corneum was not affected. The slight decreasing pattern observed could be due to an occlusive effect of the solution. The solution also caused a loss of water in the skin but with a tendency to recover after 1 h. When applying the MA gel, an increase in water loss from the stratum corneum was observed 15 min after the application, pointing to an alteration in the constitution of the skin. However, within one hour the basal values were recovered, which indicated the alterations were reversible. When using gels containing polymers with a high capacity to capture water, the skin hydration is strongly affected and water is lost from the stratum corneum. In the current study, this effect was marked at 15 min, followed by a slow gradual recuperation of hydration over the next 2 h. The application of the MA gel did not induce any visual skin irritation and was well tolerated by all the individuals participating in the study, none of whom reported pain or any inconveniences from its administration.

At the highest concentrations tested, the gel formulation with or without MA showed cytotoxicity effects on the RAW 264.7 and J774A.1 cell lines. Cytotoxicity in the macrophage cell lines was similar for all the compounds tested, indicating a similar resistance to the excipients and the MA in the formulation.

The in vitro anti-leishmanial activity on intracellular amastigotes showed the MA gel inhibited amastigote survival to a greater extent than the MA solution. Analysis of the selectivity index (SI) showed higher SI values for the MA gel formulation than for the MA solution, which indicated that the gel was less toxic and with higher activity against the parasite.

Interestingly, the amount of Sb^V^ retained in damaged and undamaged skin was greater when the gel formulation was applied compared to the solution, which makes it more advantageous for therapeutic application. These skin retention values, as well as the IC_50_ results against amastigotes (Table 3) and considering the density of the hydrated skin (0.964 g/mL) [41], indicate that the new MA gel formulation could provide high local amounts of Sb^V^, even in damaged skin, and may exert an efficient action against CL. Future experiments in an animal model need to be performed to confirm our preliminary results and determine the efficacy in vivo.

## 5. Conclusions

We have developed an easy to prepare topical MA gel-based formulation, an administration route that avoids injections and has higher patient acceptance and tolerability. It was shown to be stable for at least 6 months and has optimal properties for use in CL treatment: a biocompatible pH, easy spreadability and no skin irritant effects.

Experiments showed low permeation of the Sb^V^ through the skin, which would represent a low concentration of the drug in blood, and high retention in the skin layers. Thus, the gel formulation almost completely avoids the toxic side effects of Sb^V^ and could supply a therapeutic level of MA at the local site for an extended period.

Low cytotoxicity in the keratinocyte cell line and the two macrophages cell lines was observed. The study of anti-leishmanial activity in intracellular amastigotes treated with the MA gel showed a reduction of the IC_50_ in comparison to the reference solution. These preliminary results indicate the MA formulation could be a promising alternative for CL treatment, although further studies are required to establish a proper dosage schedule in vivo.

## Figures and Tables

**Table 1:** Concentration of Sb^V^ and SD in the reference solution and MA gel samples at the beginning of the experiment and 6 months later at different storage temperatures (t_0_ = initial time; t_180_ = after 6 months).

Formulations	Sb^V^ (µg/mL) ± SD			
	t_0_	t_180_ RT	t_180_ 37 °C	t_180_ 4 °C
MA solution	79,659.92 ± 1023.45			
MA gel	57,936.62 ± 894.92	57,436.62 ± 854.92	58,575.93 ± 654.07	59,255.83 ± 524.49

**Table 2:** Sb^V^ permeation across the skin barrier and Sb^V^ retained after 27 h in damaged and non-damaged skin.

Assay	MA Solution		MA Gel	
	Non-Damaged Skin	Damaged Skin	Non-Damaged Skin	Damaged Skin
Permeation (µg/cm^2^) ± SD	2966.50 ± 562.37	121.00 ± 59.45	1217.53 ± 279.41	774.81 ± 179.63
Retention in skin (µg/g/cm^2^) ± SD	51,672.84 ± 8964.28	2057.47 ± 381.67	71,043.69 ± 10,641.57	10,728.23 ± 2254.61

**Table 3:** In vitro activity against promastigotes and amastigotes, and cytotoxicity of the reference MA solution and the MA gel and selectivity index (SI).

Formulations (µg/mL Sb^V^)	IC_50_ (µg/mL Sb^V^ ± SD)		SI		CC_50_ (µg/mL Sb^V^ ± SD)	
	Promastigotes	Amastigotes	SI_RAW_	SI_J774_	RAW 264.7	J774A.1
MA solution	>750 (* na)	57.35 ± 2.76	6.66	6.37	381.76 ± 94.74	365.14 ± 165.26
MA gel	633.15 ± 43.26 (* na)	15.76 ± 4.81	14.19	14.03	223.66 ± 46.82	221.05 ± 65.41

* na: not active.

**Table 4:** In vitro activity against promastigotes and amastigotes, and cytotoxicity of the gel alone and selectivity index (SI).

Excipients	IC_50_ (µg/mL ± SD)		SI		CC_50_ (µg/mL ± SD)	
	Promastigotes	Amastigotes	SI_RAW_	SI_J774_	RAW 264.7	J774A.1
Gelling agents (375–0.37 µg/mL)	>375	20.39 ± 5.43	6.57	6.13	134.00 ± 42.57	125.00 ± 20.80
